# Hemodynamic changes in late advanced pregnant Zaraibi goats during the peripartum period

**DOI:** 10.1186/s12917-023-03745-7

**Published:** 2023-10-06

**Authors:** Hager Madbouly, K. H. El-Shahat, Mohamed Fathi, Elshymaa A. Abdelnaby

**Affiliations:** Theriogenology Department, Faculty of Veterinary Medicine, Cairo University, Giza, 12211 Egypt

**Keywords:** Zaraibi goats, Doppler, Umbilical artery, Maternal side, Uterine artery, Fetal heart, Ultrasonography

## Abstract

The objective of the present study was to demonstrate the blood flow velocities, blood flow rate (BFR; bpm) with the accurate ratio of both systolic and diastolic velocities points (S/D) in addition to Doppler indices (resistive and pulsatility index [RI and PI]) in both fetal [fetal heart (FH), fetal abdominal aorta (Ab. A), and umbilical artery (UM.A)] and maternal [Middle uterine artery (MU.A)] sides during the last month of gestation. Ten Zaraibi (Egyptian Nubian) goats weighing 40-50kg and aged from 5–7 years were examined twice per month till reached the last month of pregnancy. Then all females were examined every 5 days starting from day -35 till day -1 before kidding.The pregnant goats were examined by ultrasonic and Doppler indices were recorded with Doppler scanning (7.5 -12 MHz, with colored and spectral graph to form the perfect wave to assess Doppler measurements). The obtained data were analyzed using analysis of variance. Results indicated that on the fetal side; the maximum point of velocity (MSV; cm/sec) in the FH and BFRwere elevated from day -35 till day -10 with a slight decline at days -5 and -1 at the peripartum period (*P* < 0.05), while FH.PI and S/D ratio declined till day -1(*P* < 0.05). In addition, the fetal Ab. A, and UM.A PI, RI, and S/D ratio declined from day -35 till day -1 at the peripartum period with a significant increase in the peak systolic velocity (PSV) and BFR(*P* < 0.05). However, non-significant changes in the end diastolic velocity (EDV) were detected. On the maternal side, the MU.A PI and S/D declined from day -35 till day -1 with an elevation of both PSV and BFRat the same time points (*P* < 0.05). In conclusion, the Doppler evaluation of fetal and maternal blood flow vessels is important to give complete information that directly affects the health status of the mother and fetus.

## Introduction

Goats play an important role as a potential source of meat and milk, especially in developing countries. They can be raised with limited resources, and their numbers increase more quickly than those of sheep, representing an increased role in food production [1]. Goat gestation periods range from 145 to 155 days, with an average of 150 days [2]. Breed, litter weight, environment, and parity can all have an impact. Late-advanced pregnancy is the most critical period as the developing fetus gains two-thirds of its birth weight in the final six weeks of pregnancy [3]. During pregnancy, progesterone levels fluctuate; they are low from the moment of conception until the sixth day [4], then start to rise considerably by the third week and continue to rise until the 19^th^ week [5, 6]. Luteinizing hormone (LH) is necessary for the CL to continue secreting progesterone during pregnancy in goats; moreover, prolactin (PRL) and LH work together to stimulate this process [7]. The level of estradiol is observed two days before ovulation [4, 8]; it declines for the first 30 days following mating and then increases again between weeks 7 and 11, with the highest output close to parturition [6, 9]. Ultrasonography is a highly effective and accurate method for detecting pregnancy [10]. Fetal development, gestational age, and the number of fetuses in goats can all be determined using B-mode ultrasonography [11]. Doppler ultrasound is a viable, non-invasive tool to examine maternal and fetal hemodynamics throughout pregnancy. By using color and pulsed wave Doppler, information about the velocity of blood flow and blood type is measured, which has gynecological importance in animal species [12]. These Doppler ultrasound examinations in human obstetrics have proved helpful for identifying low- and high-risk pregnancies and determining the fetus's health [13, 14]. Doppler ultrasound uses resistive and pulsatility indices (RI and PI), systolic and diastolic velocities (PSV and EDV), and blood flow volume (BFV) to detect the amount of vascular perfusion [15]. More information regarding the perfusion of the fetoplacental and uteroplacental circulations, respectively, is provided by examination of the umbilical and uterine arteries [16]. Fetal cardiac activity, RI, and PI of the umbilical artery have been detected by Doppler ultrasound investigation in pregnant goats [17]. RI, PI, and peak systolic/end-diastolic (S/D ratio) are the most important Doppler indices from umbilical blood flow studies [18, 19]. As the pregnancy advances, the S/D ratio decreases [18], which means that vascular impedance decreases and vascular perfusion increase for the fetus. The peak systolic velocity (PSV) of the umbilical artery in pregnant goats increased significantly (*p* < 0.05) from day 39 to 67 and then between 98 and 120 days of gestation, while the end-diastolic velocity (EDV) was not affected [12]. Before parturition, the blood flow velocity of the fetal aorta was significantly lower, as explained by the increased aortic size with the elevation of vascular resistance of the aorta that led to a reduction of blood flow velocity and rate [12, 20]. According to the above results, the objective of this study was to demonstrate the Doppler indices (RI and PI), blood flow velocities with their ratio (PSV, EDV, and S/D ratio), and blood flow rate (BFR) in both fetal (fetal heart, fetal abdominal aorta, and umbilical artery) and maternal (Middle uterine artery) sides in the advanced critical period of gestation in Zaraibi goats.

## Materials and methods

### Ethical approval committee

This current study was accepted by the institutional animal care committee in the Faculty of Veterinary Medicine, at Cairo University with an approval number: Vet CU 01122022605.

### Experimental animals, feeding and management

The current experiment was conducted on ten (*n*=10) cyclic multiparous adult female Zaraibi (Egyptian Nubian) goats(all carried twins), aged from 5-7 years, weighing from 40-50kg, and kept on the small ruminant farm in the Theriogenology Department at the Faculty of Veterinary Medicine Cairo University (30.0276°N, 31.2101°E). Female’s diet included a pelleted ration for small ruminants, hay with free access to water with salt all day according to NRC requirements [21].Animals were not euthanized after the end of the examination, but their kept until kidding, as we performed a future research at their puerperium period. Goats were housed in sheds with mud floor with fodder trees can be grown around the shed, which acts as a source of feed for the growing goats , and clean drinking water should be available for goats. The examination period was October to November 2022.

### Examination, estrous synchronization, and pregnancy diagnosis

All females were examined in form of routine monitoring of the pulse rate, respiratory rate, rumen activity, and heart rate. Only normal females without any problems especially cardiovascular diseases were used in this experiment [22]. Females were synchronized by Ovsynch protocol [(intramuscular administration of gonadorelin (50 mg; C55H75N17O13, CID 638793 synthetic GnRH), then another intramuscular administration of cloprostenol (125 mg; seven days later; ESTRUMATE), then after 2 days another dose of gonadorelin was administrated (50 mg; C55H75N17O13, CID 638793 synthetic GnRH [23]). Then all females were mated with an excellent fertile buck twice after the standing reflex and then 12 hours later. In all females, the insemination and last mating date were recorded as day 0. All females were diagnosed for the achievement of pregnancy as early as 25 days which revealed an enlargement of the uterine lumen with the presence of amniotic fluid within embryonic mass and heartbeats. Animals were examined twice/month till reached the last month of pregnancy then all females were examined every 5 days starting from day -35 till day -1 before kidding.

### Ultrasonography (B-mode and Doppler)

**Fig. 1:**
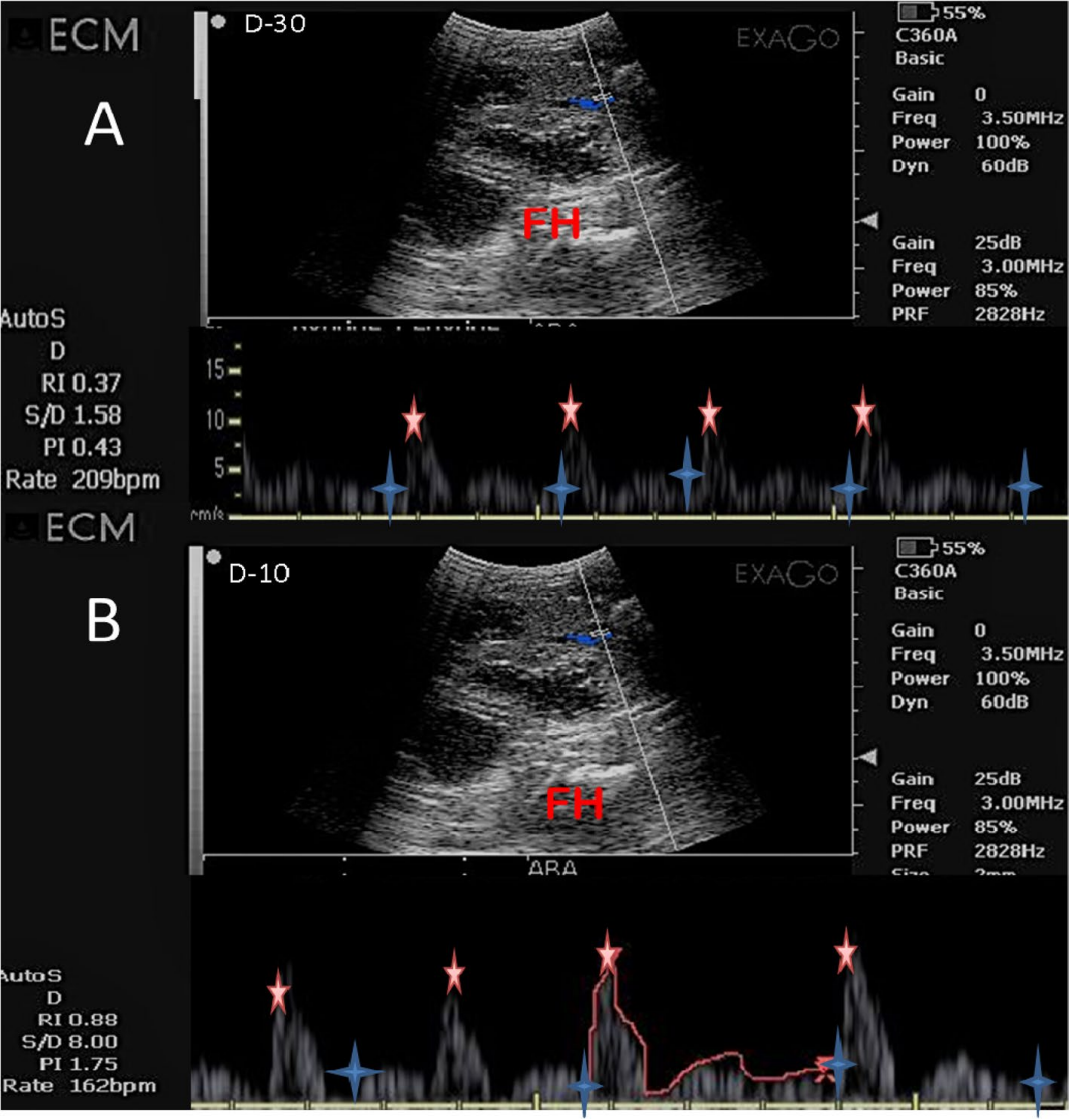
Pulsed wave Doppler ultrasonographic images of the fetal heart (FH) at days [(-30; **A**), and -10 (**B**)] before parturition in a *Zaraibi goat* with the automatic calculation of both Doppler indices (RI and PI), S/D ratio, and BFR(bpm). The red star showed the maximum systolic point of velocity (MSV; cm/sec), while the blue asterisk showed the end point of velocity due to relaxation (EDV; cm/sec). RI = resistive index, PI = pulsatility index, S/D = systole/diastole, and BFR = blood flow rate

**Fig. 2:**
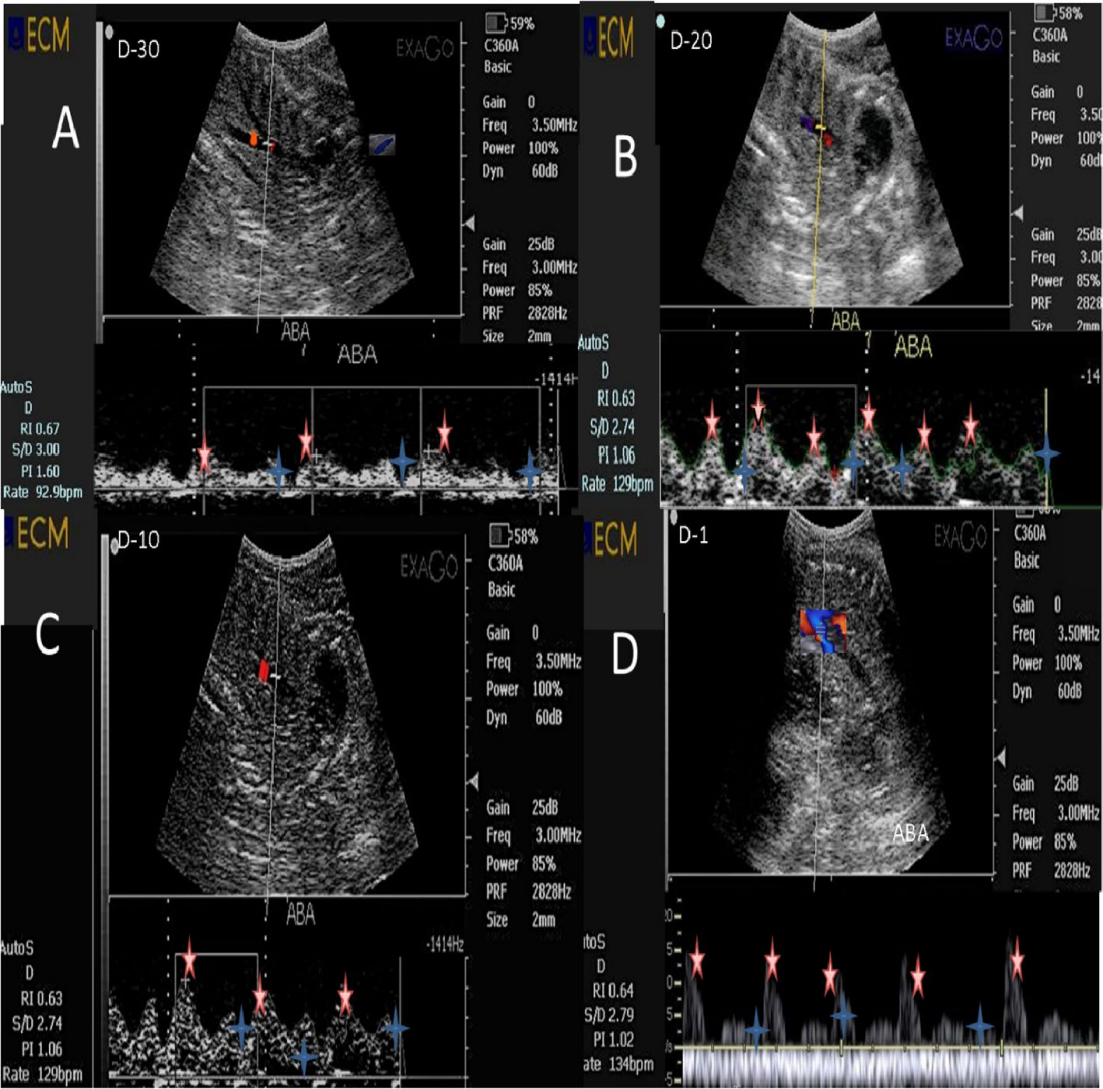
Pulsed wave Doppler ultrasonographic images of the fetal abdominal aorta (Ab.A) at days [(-30; **A**),-20 (**B**), -10 (**C**), and (-1; **D**)] before parturition in a *Zaraibi goat* with the automatic calculation of both Doppler indices (RI and PI), S/D ratio, and BFR(bpm). The red star showed the maximum systolic point of velocity (MSV; cm/sec), while the blue asterisk showed the end point of velocity due to relaxation (EDV; cm/sec).RI = resistive index, PI = pulsatility index, S/D = systole/diastole, and BFR = blood flow rate

The routine ultrasonographic assessment was performed first by B-mode and then by Doppler mode activation by using the EXAGO Doppler ultrasound device (EXAGO, Echo Control Medical, Angoulême, France) with a frequency of 7.5 -12 MHz equipped with a convex probe, the assessment was done while animals in a standing position, and was adequately restrained by the assistant, all measures were taken by only the same operator. The ultrasound scanning was positioned on the right and left of the inguinal area and moves ventrally in the abdominal area after trimming all hair and applying adequate gel in order to minimize any artifacts present due to the air medium [24].The fetal heart (FH) and fetal abdominal aorta (Ab. A) were located easily using first B-mode, then after activation of the color Doppler mode the FH and Ab. A were visualized within colors red and blue that determine the direction of the blood (Figs. 1 and 2) Using spectral Doppler mode in order to activate the gate entered into the FH and Ab .A to detect both Doppler indices expressed by resistive and pulsatility indices (RI and PI) and blood flow velocity expressed by peak systolic and end diastolic velocities (PSV and EDV; cm/sec), and blood flow rate of the specific vessel (BFR) measured in beat per minute (bpm) as shown in (Figs. 1 and 2). The systolic /diastolic ratio (S/D) could provide important information about the amount of blood flow velocity entered in the known vessel on the maternal and fetal side.

**Fig. 3:**
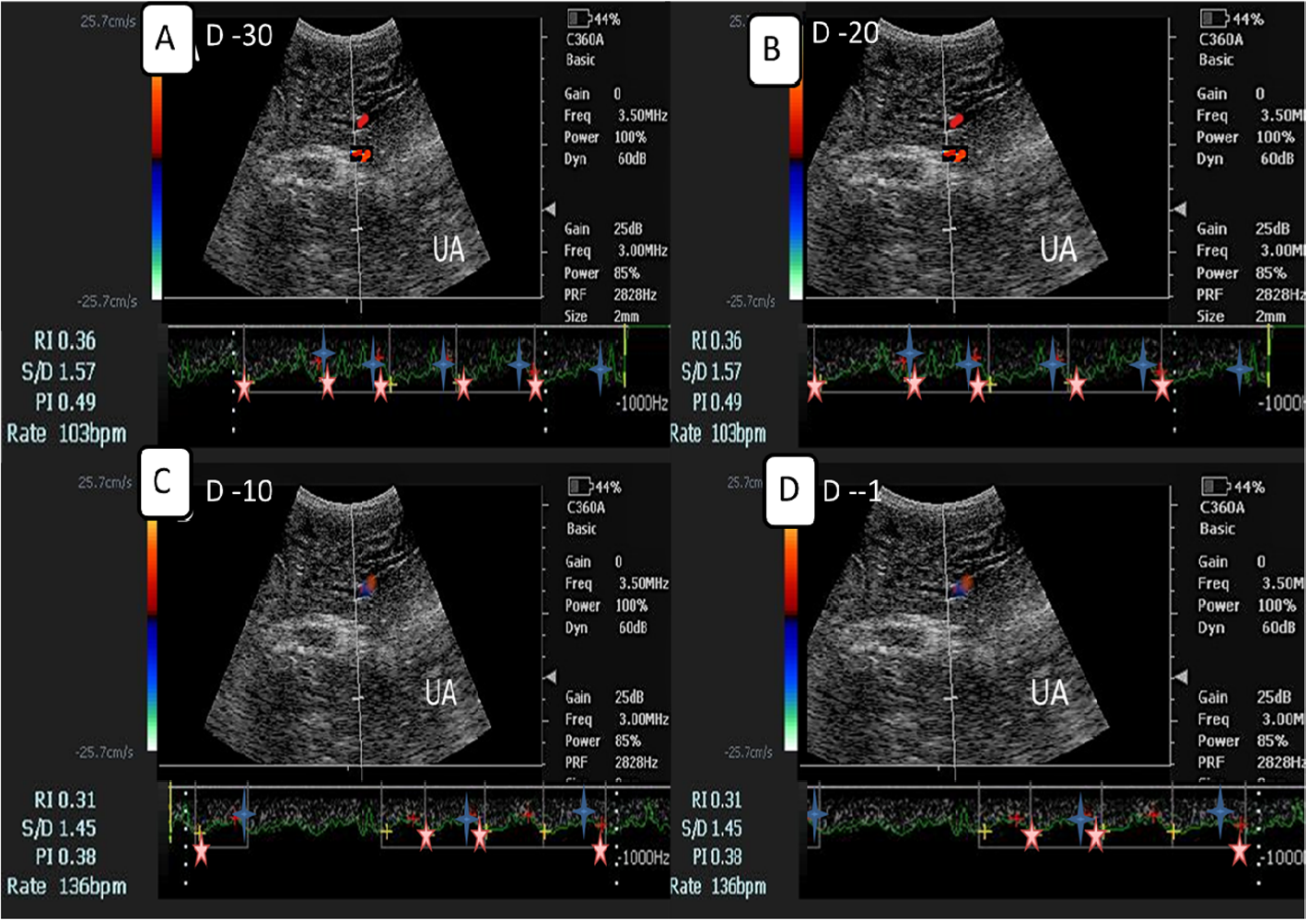
Pulsed wave Doppler ultrasonographic images of the dam middle uterine artery (MUA at days [(-30;**A**),-20(**B**), -10(**C**), and (-1;**D**)] before parturition in aa Zaraibi goats with the automatic calculation of both Doppler indices (RI and PI), S/D ratio, and BFR(bpm).Red star showed the maximum systolic point of velocity (MSV; cm/sec),while the blue asterisk showed the end point of velocity due to relaxation (EDV; cm/sec).RI = resistive index, PI = pulsatility index,S/D = systole/diastole, and BFR = blood flow rate

The middle uterine artery (MUA; Fig. 3) was assessed after locating the urethral artery that supplies the urinary bladder, then the uterine artery was merged from the internal iliac artery and the flow was measured once detected craniolaterally [19], while umbilical artery (UMA) was detected freely foliating within umbilical cord [17] but not easy to be detected as MUA. After locating each artery using the color mode, the pulsed wave Doppler was activated with 1mm gate thickness, 35 cm/sec maximum velocity adjusted by the device, and angle of insonation 40° [25].

### Statistical analysis

The presented results were obtained using SPSS software version 20 (Microsoft Corp. 1984–2000 Inc.), using ANOVA options by one- way method to show alterations in each variable all over the examined time points. the data were presented as mean and standard error(SEM) Duncan's multiple range test was used as a means to detect the significant difference at a probability less than 0.05.

## Results

### Fetal side peripartum alterations

#### The hemodynamics changes in the fetal heart (FH)

**Fig. 4:**
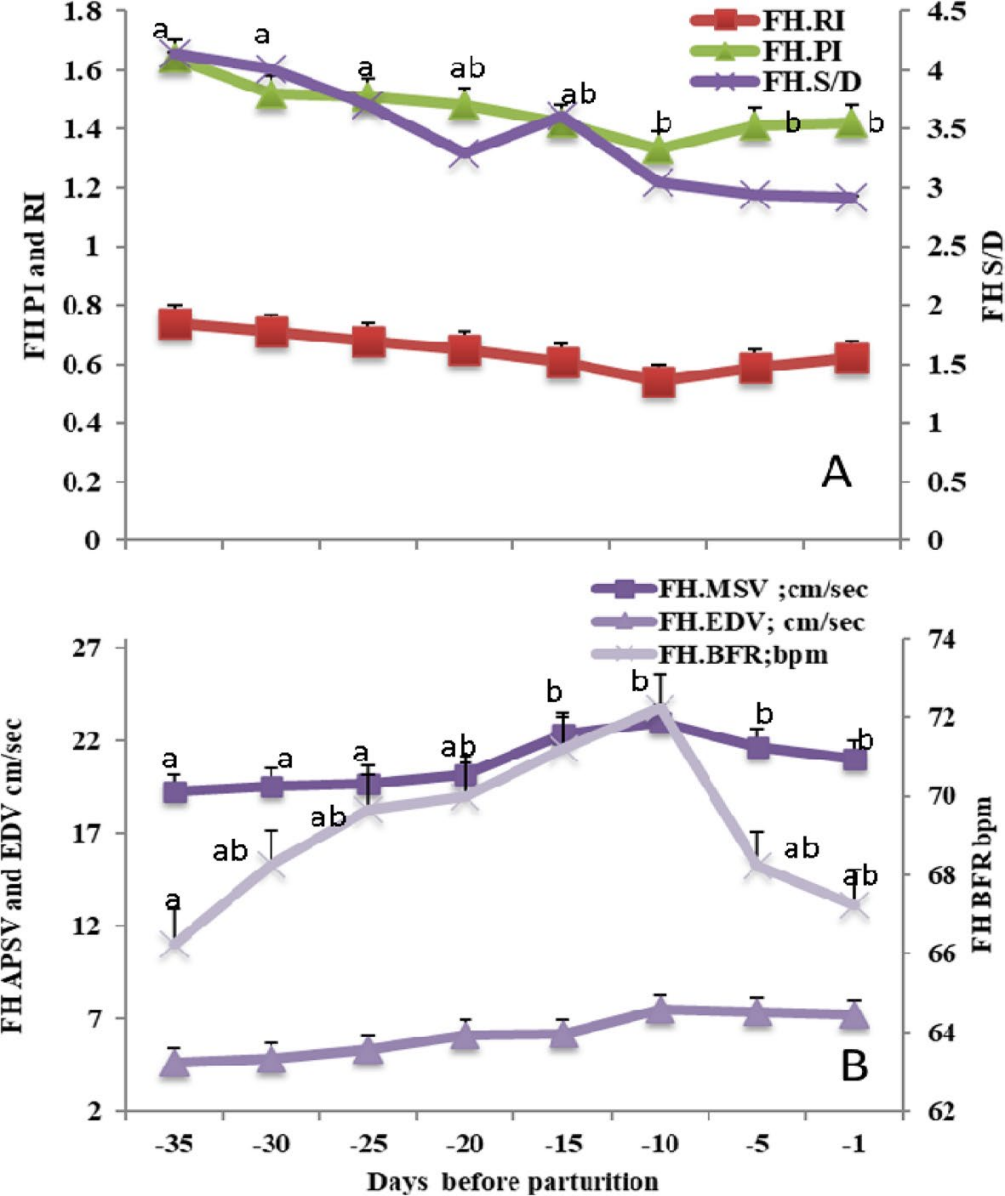
Mean ± standard error of mean (SEM) of the fetal heart (FH) resistive index (FH.RI on the primary axis), pulsatility index (FH.PI on the primary axis), and systolic/diastolic ratio (FH.S/D on the secondary axis; **A**), in addition to fetal heart maximum systolic velocity (FH.MSV; cm/sec), end velocity (FH.EDV; cm/sec) and blood flow rate (FH.BFR; bpm; **B**) in *Zaraibi* goat in the peripartum period (from day -35 till day -1). Means with different letters means there was a significant difference at a probability less than 0.05 (*P* < 0.05)

Both FH.PI and S/D ratio were significantly (*P*<0.05) declined from day -35 till day -1 before parturition with a slight elevation of PI at days -5 and -1 before birth. While, FH.RI was declined gradually with non-significant all over peripartum days (Fig. 4A). Noticeable changes occurred in blood flow velocity and blood flow rate (BFR), with significantly (*P*<0.05) maximum point of velocity (MSV; cm/sec) and BFR (bpm) were observed from day -35 till day -10. Then, a slightly marked decline at days -5 and -1 for both parameters, while the end point of velocity (EDV) was not affected (Fig. 4B).

#### The hemodynamics changes in the fetal abdominal aorta (Ab. A)

**Fig. 5:**
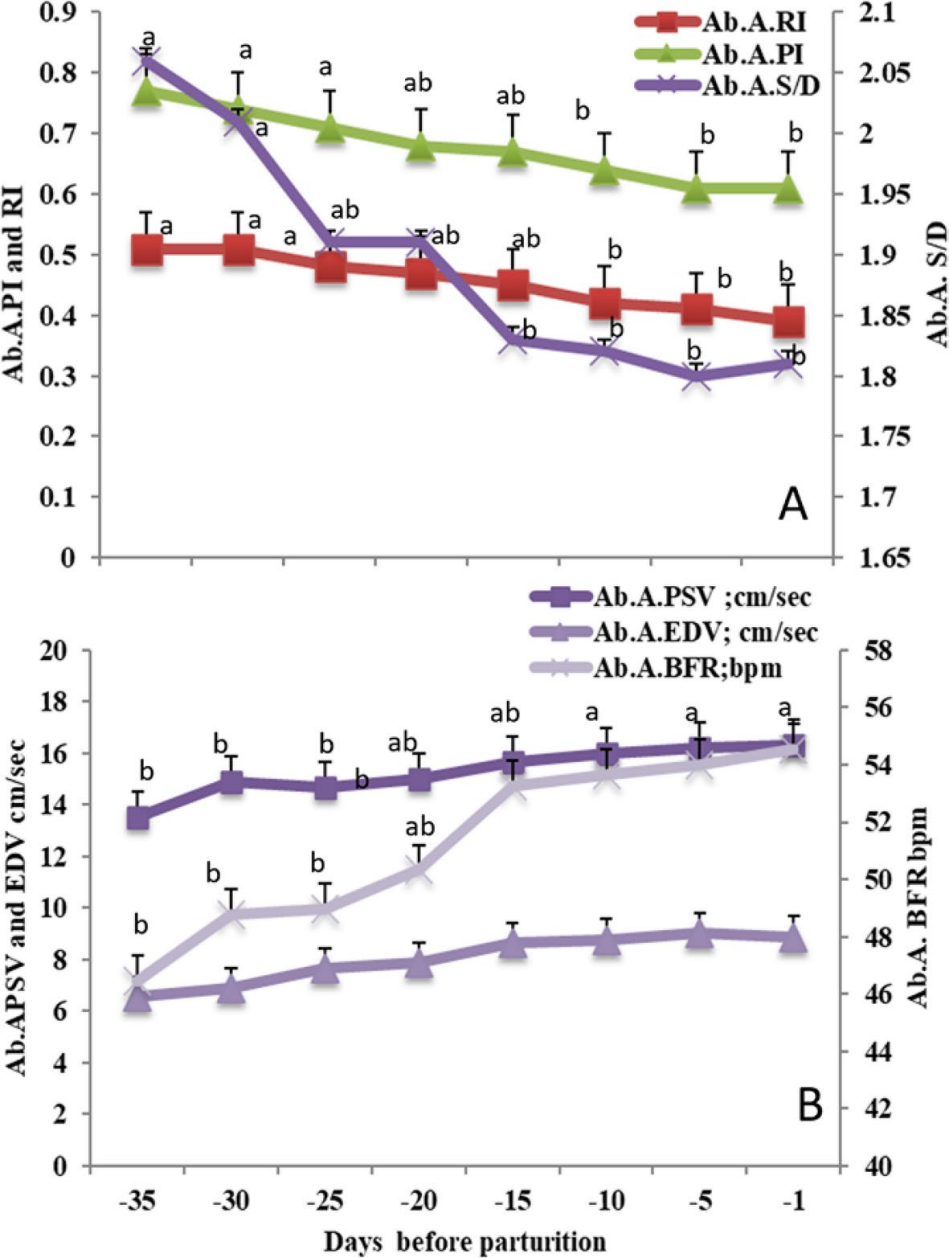
Mean ± standard error of mean (SEM) of the fetal abdominal aorta (Ab. **A**) resistive index (Ab. A. RI on the primary axis), pulsatility index (Ab. A. PI on the primary axis), and systolic/diastolic ratio (Ab. A. S/D on the secondary axis; A), in addition to fetal Ab. A. peak systolic velocity (Ab. A. PSV; cm/sec), end velocity (Ab. A. EDV; cm/sec) and blood flow rate (Ab. A. BFR; bpm; **B**) in *Zaraibi* goat in the peripartum period ( from day -35 till day -1). Means with different letters means there was a significant difference at a probability less than 0.05 (*P* < 0.05)

Ab. A PI, RI, and S/D ratio significantly (*P*<0.05) declined from day -35 till day -1 at the peripartum period (Fig. 5A). Noticeable changes occurred in peak velocity with the rate, peak systolic point of velocity (PSV), and blood flow rate (BFR) were significantly (*P*<0.05) elevated from day -35 till day -1 at the peripartum period with the non-significant changes in EDV (Fig. 5B).

#### The hemodynamics changes in the fetal umbilical artery (UM. A)

**Fig. 6:**
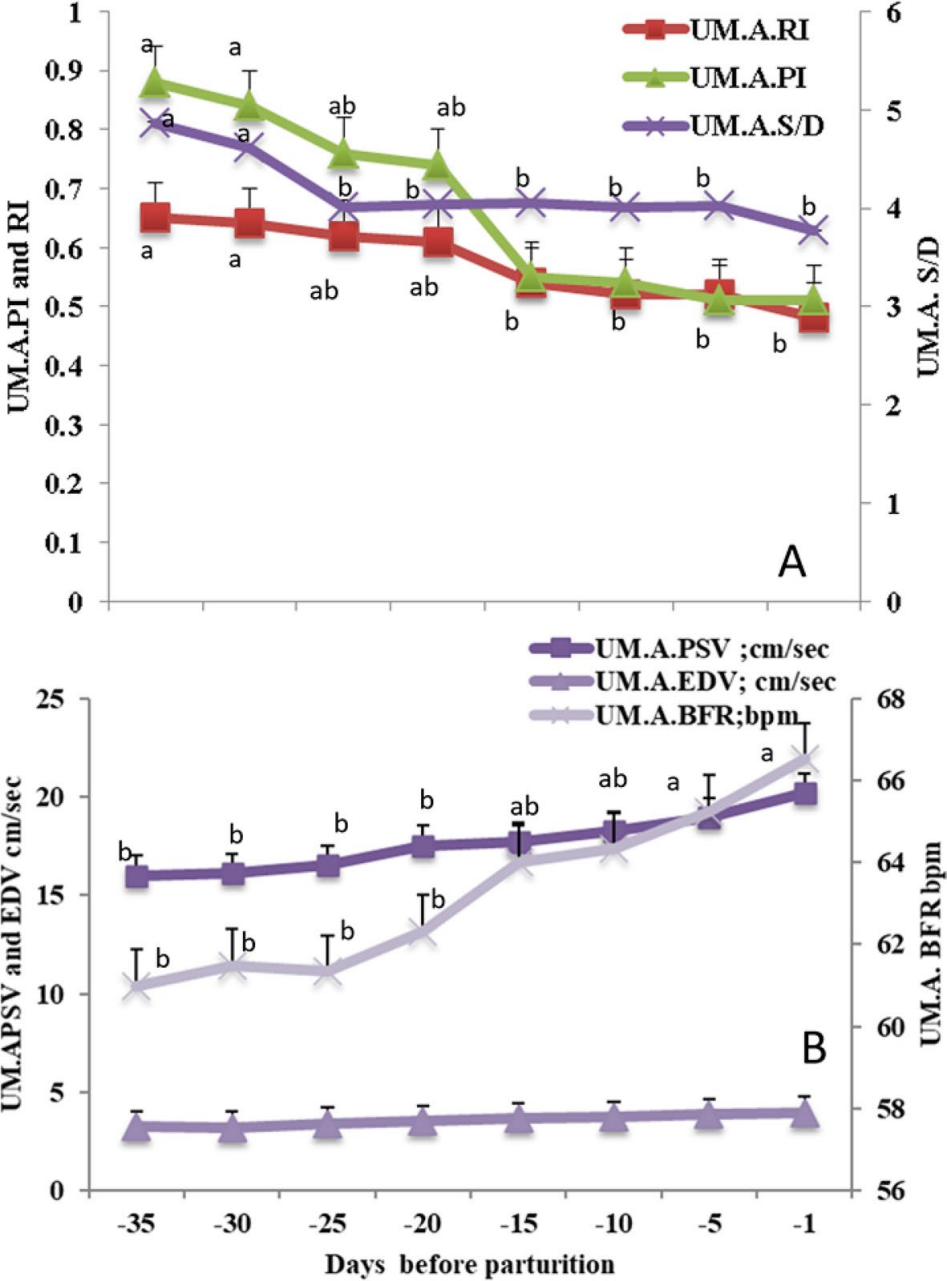
Mean ± standard error of mean (SEM) of the fetal umbilical artery (UM. A) resistive index (UM. A. RI on the primary axis), pulsatility index (UM. A. PI on the primary axis), and systolic/diastolic ratio (UM. A. S/D on the secondary axis; **A**), in addition to peak systolic velocity (UM.A. PSV; cm/sec), end velocity (UM.A. EDV; cm/sec) and blood flow rate (UM.A. BFR; bpm; **B**) in *Zaraibi* goat in the peripartum period ( from day -35 till day -1). Means with different letters means there was a significant difference at a probability less than 0.05 (*P* < 0.05)

The UM. A S/D ratio, and both Doppler indices (RI and PI) significantly (*P*<0.05) declined from day -35 till day -1 at the peripartum period (Fig. 6A). In addition, PSV and BFR were significantly (*P*<0.05) increased at the same time points while EDV was not affected (Fig. 6B).

### Maternal side peripartum alterations

**Fig. 7:**
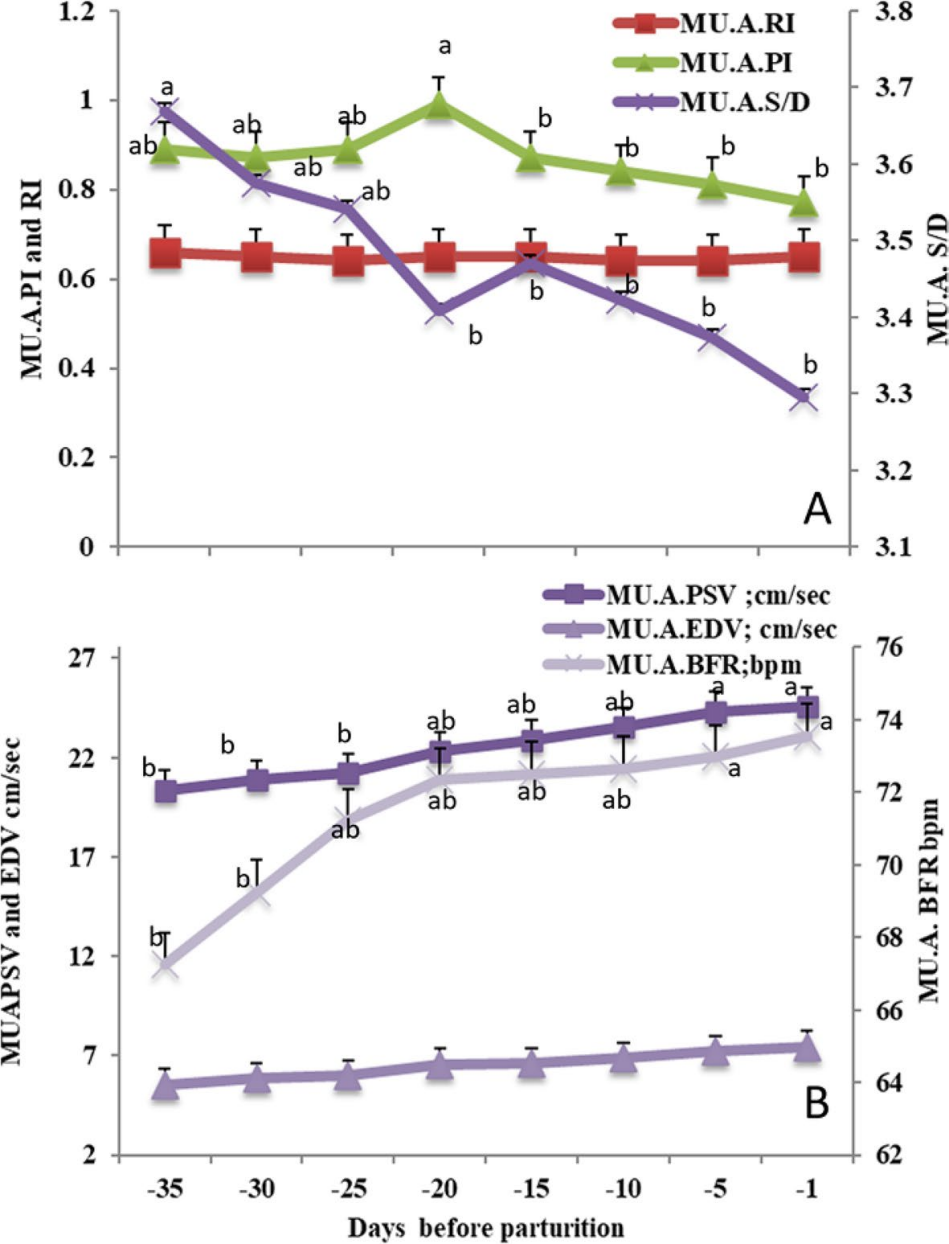
Mean ± standard error of mean (SEM) of the maternal middle uterine artery (MU.A.) resistive index (MU. A. RI on the primary axis), pulsatility index (MU. A. PI on the primary axis), and systolic/diastolic ratio (MU. A. S/D on the secondary axis; **A**), in addition to peak systolic velocity (MU.A. PSV; cm/sec), end velocity (MU.A. EDV; cm/sec) and blood flow rate (MU.A. BFR; bpm; **B**) in *Zaraibi* goat in the peripartum period (from day -35 till day -1). Means with different letters means there was a significant difference at a probability less than 0.05 (*P* < 0.05)

The maternal side was expressed by alterations in the middle uterine artery (MU.A) that supplies the uterus, as the Doppler index PI was only affected while the RI was not changed in any form (Fig. 7A), the PI and S/D ratio declined (*P*<0.05) from day -35 till day -1 at the peripartum. In addition the PSV and BFR of the MU.A were increased (*P*<0.05) from day -35 till day -1 at the peripartum, with no detectable changes in EDV(cm/sec) as shown in (Fig. 7B).

## Discussion

The present findings revealed that it was imaginable to demonstrate the Doppler indices (RI and PI), blood flow velocities with their ratio (PSV, EDV, and S/D ratio), and blood flow rate (bpm) in both fetal (FH, Ab. A, and UM.A), and maternal (MU.A) sides. The present work provided important data related to a good understanding of the advanced critical period of gestation vascularity in goats. The MUA could be assessed easily without any problems all over the gestational months, but the UM.A is closely related to gestational age. A similar study evaluated the fetal heart with the abdominal aorta at the second and third stages of pregnancy in Bulgarian white milk goats [20]. In addition, the umbilical and uterine arteries were also recorded in Saanen goats [26]. However, the present study was the first to demonstrate the blood flow rate with the accurate ration of both systolic and diastolic points (S/D) in addition to Doppler indices at the last month of gestation with accurate measurements of alterations in blood flow parameters every 5 days until day -1 before birth. The FH MPV, and BFR were elevated at the last month of gestation with a marked decline observed a few days before kidding. This could be explained by the need for nutrients and oxygen all over the last month in order to give fetus at the optimum performance [27] that lead to an increase in the blood supply and blood flow velocity with the reduction of both Doppler indices, and S/D due to the inverse relationship between both parameters [25, 28, 29]. On other hand, the reduction in blood flow a few days before kidding accompanied by the elevations of both Doppler indices and S/D at the same time. This may be attributed to that the fetus is not able to adapt to this condition with lack of nutrients and space and this could enhance fetus to initiate the process of parturition by elevating the fetal cortisol due to the stressful factors [30].

The reduction of RI and PI recorded herein at gestational months in Ab A, UM.A and FH could be a mark for the normal pattern that revealed the elevation of blood flow velocities especially the peak systolic velocity (PSV). Some studies evaluated the reduction RI in UMA, AB aorta, and fetal renal arteritis in the woman and veterinary medicine [31, 32] and monitored the alterations happens to get a references value to the hemodynamics at this period. In accordance with our results, a study reported that Doppler indices reduction was related to the state of smaller vessels downstream from the examined artery [32]. As this could be contributing to the blood flow resistance [16, 33]. Those Doppler indices values were originally obtained from the peak point and end point of velocity in addition to the time mean to obtain successive cardiac cycles in the spectral graph [23]. Contrary some studies revealed, the reduction of the resistive index was accompanied by unfavorable outcomes [34, 35]. The blood flow velocity of the fetal aorta was significantly lower before parturition, as explained by the increased aortic size with the elevation of vascular resistance of the aorta that led to a reduction of blood flow velocity and rate [12, 20].

The MUA assessment by pulsed wave Doppler during the later stage of pregnancy is very important to detect any abnormalities as intrauterine diseases [26]. According to the authors, the uterine RI and PI were elevated from 8–20 weeks of gestation and then declined gradually till the late stage of pregnancy, but maternal anxiety could play a role that may affect the accuracy of the results due to the bad temperament of the mother and movements of animals that could adversely affect the perfect wave [36]even after parturition in some cases[37], while the free foliating UMA in the fetus could be easily detected even in the later stage of pregnancy with the estimation of its blood flow velocities that give a good prediction on the fetus' health and performance after birth by measuring the amount of PSV, S/D, and BFR that gives a good image about the functional status of the fetus in early pregnancy [38], even when twins are present [39], all goats carrying twins showed a greater value of PI and RI in their UM.A compared to AB.A and FH, but this high value decreased during the last few months, which directly affects the oxygen demands and nutrient requirements needed by the two feti [40, 41].

As a limitation of this study, we determine the Doppler indices of the umbilical artery by using the rectal probe of 7.5MHz frequency during the last month of gestation by passing in the rectum in order to easily detect the free foliating artery which limits the device depth and gain being crucial at this late stage of pregnancy.

## Conclusion

The present study is the first report recorded the hemodynamic alterations occurred in both fetal and maternal sides every 5 days till the day before kidding process. The study both fetal and maternal blood flow vessels is important to give a complete information that directly affects the mother and the fetus Its predictable that those data contribute to understanding the normal hemodynamic pattern at this last critical period and compare the Doppler measurements values to be taken as reference range in order to estimate any abnormalities.

## Data Availability

All data collected or analyzed during this study are included in this published paper.
